# Predicting the Progression from Asymptomatic to Symptomatic Multiple Myeloma and Stage Classification Using Gene Expression Data

**DOI:** 10.3390/cancers17020332

**Published:** 2025-01-20

**Authors:** Nestoras Karathanasis, George M. Spyrou

**Affiliations:** Bioinformatics Department, The Cyprus Institute of Neurology & Genetics, 6 Iroon Avenue, Ayios Dometios, 2371 Nicosia, Cyprus; georges@cing.ac.cy

**Keywords:** multiple myeloma, cancer, gammopathies, progression, machine learning

## Abstract

Background: The accurate staging of multiple myeloma (MM) is essential for optimizing treatment strategies, while predicting the progression of asymptomatic patients, also referred to as monoclonal gammopathy of undetermined significance (MGUS), to symptomatic MM remains a significant challenge due to limited data. This study aimed to develop machine learning models to enhance MM staging accuracy and stratify asymptomatic patients by their risk of progression. Methods: We utilized gene expression microarray datasets to develop machine learning models, combined with various data transformations. For multiple myeloma staging, models were trained on a single dataset and validated across five independent datasets, with performance evaluated using multiclass area under the curve (AUC) metrics. To predict progression in asymptomatic patients, we employed two approaches: (1) training models on a dataset comprising asymptomatic patients who either progressed or remained stable without progressing to multiple myeloma, and (2) training models on multiple datasets combining asymptomatic and multiple myeloma samples and then testing their ability to distinguish between asymptomatic and asymptomatic that progressed. We performed feature selection and enrichment analyses to identify key signaling pathways underlying disease stages and progression. Results: Multiple myeloma staging models demonstrated high efficacy, with ElasticNet achieving consistent multiclass AUC values of 0.9 across datasets and transformations, demonstrating robust generalizability. For asymptomatic progression, both modeling approaches yielded similar results, with AUC values exceeding 0.8 across datasets and algorithms (ElasticNet, Boosting, and Support Vector Machines), underscoring their potential in identifying progression risk. Enrichment analyses revealed key pathways, including PI3K-Akt, MAPK, Wnt, and mTOR, as central to MM pathogenesis. Conclusions: To the best of our knowledge, this is the first study to utilize gene expression datasets for classifying patients across different stages of multiple myeloma and to integrate multiple myeloma with asymptomatic cases to predict disease progression, offering a novel methodology with potential clinical applications in patient monitoring and early intervention.

## Simple Summary

Multiple myeloma is a blood cancer that progresses through distinct stages, and identifying these stages accurately is crucial for selecting effective treatments. Additionally, understanding which individuals with an asymptomatic precursor condition, known as monoclonal gammopathy of undetermined significance, are at risk of developing full-blown multiple myeloma remains a significant challenge. This study used machine learning methods to analyze gene expression data from multiple datasets, aiming to improve the accuracy of disease staging and identify individuals at higher risk of progression. By finding key patterns and pathways involved in the disease, this research offers new tools for earlier intervention and personalized care. These findings could significantly benefit the research and medical communities by improving diagnosis, enhancing patient monitoring, and opening avenues for targeted therapies.

## 1. Introduction

Multiple myeloma constitutes approximately 1% of all cancer cases and about 10% of hematologic malignancies [1,2]. Annually, more than 32,000 new cases are diagnosed in the United States, with nearly 13,000 resulting in fatalities [3]. The yearly age-adjusted incidence has remained steady for decades, hovering around 4 per 100,000 individuals [4]. It shows a slight preference for men over women and is twice as prevalent among African Americans compared to Caucasians [5]. The median age at diagnosis is typically around 65 years [6].

Nearly all multiple myeloma patients progress from an asymptomatic precursor stage known as monoclonal gammopathy of undetermined significance (MGUS) [7,8]. MGUS is found in roughly 5% of individuals aged over 50, with a prevalence around twice as high among Blacks compared to Whites [9,10,11,12]. MGUS transitions to multiple myeloma or related malignancies at a rate of 1% per year [13,14]. As MGUS is asymptomatic, over 50% of those diagnosed with it have likely harbored the condition for over a decade before clinical diagnosis [15]. In particular cases, an intermediate asymptomatic but more advanced pre-malignant stage, termed smoldering multiple myeloma (SMM), may be clinically recognizable [16]. SMM progresses to multiple myeloma at a rate of approximately 10% per year within the first five years post diagnosis, followed by 3% annually over the subsequent five years and 1.5% per year thereafter. This progression rate is influenced by the underlying cytogenetic profile, with patients harboring specific translocations at a higher risk of progressing from MGUS or SMM to multiple myeloma [17,18,19].

Despite notable therapeutic advancements in recent years, multiple myeloma (MM) remains an uncurable disease. Enhanced insights into MM’s biology and pathogenesis have prompted a transformative shift in managing MM and its precursor states, monoclonal gammopathy of undetermined significance (MGUS) and smoldering multiple myeloma (SMM) [20]. The conventional notion that MM treatment should only start upon the onset of symptoms has been challenged by the introduction of novel therapies characterized by both safety and efficacy. Clinical trials have underscored the significance of initiating treatment early in high-risk asymptomatic cases, demonstrating a marked delay in disease progression and improved progression-free survival outcomes for patients [21,22].

Yet, a critical challenge persists in identifying individuals with asymptomatic myeloma at the highest risk of progression, thereby maximizing the benefits of early treatment strategies. While risk stratification models such as the Mayo Clinic model [23] and the Spanish model [24] have been valuable, they still possess notable limitations, particularly in the context of modern therapies. Studies have revealed that patients with high-risk cytogenetic MM, including del17p, t(4;14), or t(14;20), may achieve survival rates comparable to standard-risk patients through intensified treatment regimens involving a combination of proteasome inhibitors, immunomodulatory drugs, and autologous stem cell transplantation [25]. Consequently, there is an urgent imperative to deepen our comprehension of the molecular mechanisms underpinning disease progression and refine risk stratification models for asymptomatic MM concurrently with endeavors to optimize early treatment strategies.

Over the past several years, there has been a notable increase in the utilization of machine learning (ML) algorithms and deep learning (DL) procedures for tumor detection. These methods leverage diverse data sources, including proteomic, genomic, histopathological data, and images, as well as blood and biochemical exams. Such techniques have proven beneficial not only in the realm of solid tumors but also in the management of hematological malignancies. Recent studies on multiple myeloma have emphasized the role of ML in diagnosing the disease through blood and biochemical exams and identifying bone lesions through image data. Furthermore, ML applications have been utilized to predict prognosis and therapeutic responses in multiple myeloma by analyzing gene expression data, highlighting their growing importance in personalized treatment strategies for hematologic cancers [26].

Currently, serum markers are employed to categorize MGUS patients into different clinical risk groups. However, no established molecular signature can reliably predict the progression of MGUS. To address this gap, Sun et al. [27] conducted a study utilizing gene expression profiling to stratify the risk of MGUS and devised a signature based on extensive samples with long-term follow-up. They analyzed microarrays of plasma cell mRNA from 334 MGUS patients with stable disease and 40 MGUS patients who progressed to multiple myeloma (MM) within a decade and identified a thirty-six-gene molecular signature indicative of MGUS risk.

The objectives of this study are as follows: (1) Develop machine learning models capable of accurately predicting the stage of multiple myeloma (MM) based on microarray datasets. We utilized advanced algorithms to analyze molecular data to classify patients into different stages of the disease, thereby aiding clinicians in making more informed treatment decisions. (2) By leveraging microarray datasets containing gene expression profiles and clinical information from patients in the MGUS stage, we developed predictive models that identify individuals at high risk of progressing to MM. Models trained to distinguish *MGUS from MM* were tested for their effectiveness in separating *MGUS from progressing MGUS* cases [27], with results indicating similar or better performance to models explicitly trained for this task. This proactive approach aims to enable early intervention strategies and improve patient outcomes by potentially delaying or preventing disease progression. It is important to note that the use of microarray data was a mandate for this task, as, to the best of our knowledge, no other omics data currently exist that include MGUS and progressing MGUS samples.

## 2. Materials and Methods

### 2.1. Source of Microarray Datasets and Description of Data Variables and Features

We downloaded seven microarray datasets, two from ArrayExpress and five from the Gene Expression Omnibus. In all cases, the samples were CD-138+ bone marrow plasma cells from patients with different stages of multiple myeloma (MGUS, MM) and healthy. The datasets came from four different platforms (A-AFFY-33, A-AFFY-44, GPL96, GPL570) and contained different numbers of patients in total and per stage (see Table 1).

For each dataset, we downloaded the raw .cel files. We calculated the expression matrix using the “Robust Multi-Array Average” expression measure via the rma() function of the affy or oligo R packages, depending on the requirements of each dataset, with background correction. At this step of the analysis, data were not normalized. Datasets from different platforms had different numbers of probes. GLP96 and A-AFFY-33/A-AFFY-34 contained ~22,000 probes, whereas GLP570 and A-AFFY-44 contained ~55,000. We retained only the 22,277 shared probes across all datasets. Also, datasets contained samples corresponding to disease stages outside of the scope of this study (for example, SMM, relapse MM, PCL, and HUVEC), which we removed from our analysis.

### 2.2. Data Cleaning and Preprocessing Techniques

**Figure 1:**
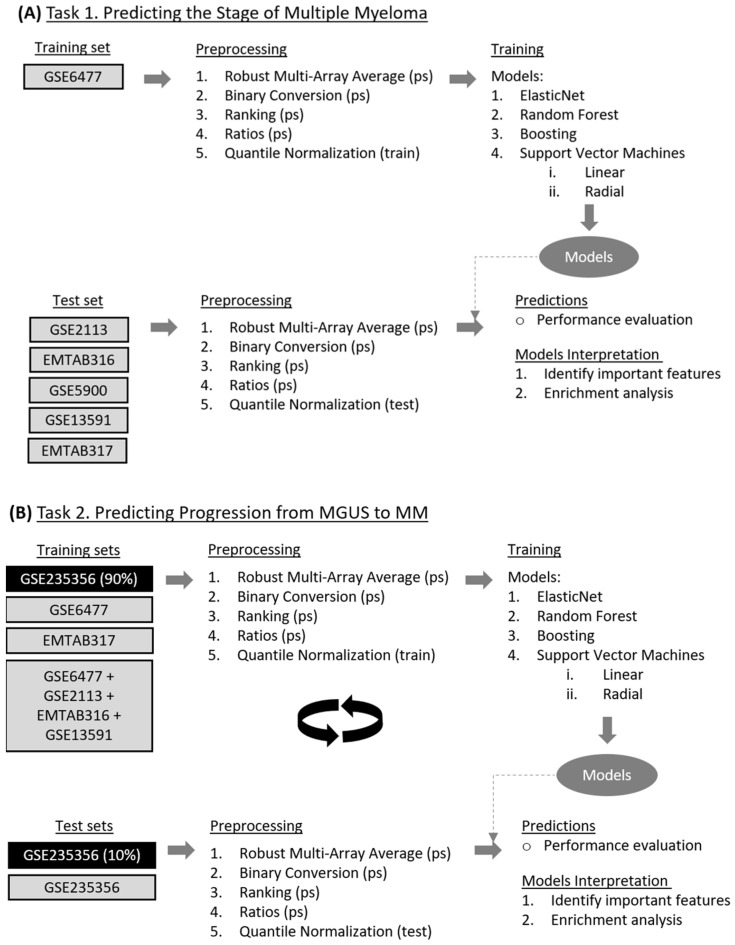
Flowchart of the analysis. (**A**) The flowchart illustrates the process used for predicting the stage of multiple myeloma. The method encompasses multiple steps: data preprocessing, model training, and performance evaluation applied across various datasets. Preprocessing includes several data transformations and the training phase incorporates a variety of machine learning models. After predictions, the model’s key features were interpreted through enrichment analyses. In the figure, (ps) indicates per-sample preprocessing, (train) indicates that normalization was applied to training samples, and (test) refers to applying the parameters learned from training to the test set. (**B**) The flowchart outlines the process used for predicting the progression of MGUS to MM using machine learning techniques. The method involves preprocessing, model training, and performance evaluation using different datasets similar to A. The boxes with a black background indicate the use of the GSE235356 dataset for training and testing in a 10-fold nested cross-validation fashion. In contrast, gray background boxes represent training on various datasets and testing on the GSE235356 dataset.

We employed several data transformation and normalization techniques to prepare our datasets for analysis (refer to Figure 1):

Robust Multi-Array Average (rma): We utilized the rma function with data background correction, which was implemented in the affy or oligo R packages, depending on the requirements of each dataset.

Binary Conversion: Expression values from rma were converted to binary (0–1) using two quantile thresholds, 0 (binary_0) and 0.5 (binary_0.5) per sample. Values exceeding the quantile threshold were set to 1, while those equal to or below the threshold were set to 0. In the case of binary_0, all values except the minimum were set to 1, and the minimum value was set to 0. Binary_0 was used as a negative control, where we expected the machine learning algorithms to perform as random classifiers, offering a baseline for performance comparison.

Ranking (ranking): Expression values were ranked from 0 to 1, with the highest value assigned a rank of 1. This ranking system provided a relative measure of gene expression levels within each sample.

Ratios (ratio): We selected only healthy samples from the GSE6477 dataset, which served solely as a training set. We calculated the ratios by performing the following steps. First, we used the ranks from the ranking transformation and calculated the standard deviation of each probe. We kept 210 probes with the lowest standard deviation. This number was chosen to minimize feature combinations, as the total number of combinations when selecting two genes each time was 21,945 features—close to the total number of features from the other preprocessing approaches. Then, we calculated all possible ratios of these probes.

Quantile Normalization (qnorm): Quantile normalization was applied in a train–test fashion using the preprocess R package. The training set underwent quantile normalization, and the parameters learned from this process were then applied to the test set. This approach ensured consistency in data distribution between the training and test datasets.

### 2.3. Overview of Machine Learning (ML) Algorithms

We evaluated the following parametric and non-parametric methods (see Figure 1).

ElasticNet (glmnet) is a parametric method that fits generalized linear and similar models via penalized maximum likelihood [28]. We employed its implementation in the glmnet package in R. ElasticNet’s advantage is that it is the most interpretable ML method [29] among these mentioned here.

Random Forest (rf) is a non-parametric tree-based method. We utilized its implementation in the randomForest R package. RF is somewhat interpretable as it provides information on which features are more important for the model by calculating variable importance scores [29].

Boosting (gbm) is a non-parametric method. We used gradient boosting machines implemented in the gbm package in R. Like RF, boosting is somewhat interpretable and provides the most important features [30].

Support Vector Machine (SVM) is a non-parametric method that has the advantage of projecting the data to a different feature space [29]. However, even though SVMs can produce very accurate models, they lack interpretability. We used the implementation of SVMs in the e1071 [31] R package to fit an SVM with the linear kernel (svmLinear2) and the implementation in the kernlab [32] R package to fit an SVM with the radial kernel (svmRadial).

### 2.4. Model Training and Interpretation

We utilized the caret R package, which stands for classification and regression training [33], to train, optimize, and test our models (see Figure 1). In order to optimize the model’s hyperparameters, we employed a ten-fold cross-validation repeated ten times. As the performance metric to determine the best model, we used the multiclass area under the ROC curve (multiclass_auc) for multiclass problems (see task 1 below) and the area under the ROC curve (AUC) for two-class problems (see task 2 below). For all models, except svmRadial, we tuned our models in a set of ten hyperparameters by setting caret’s tuneLength argument to ten. For the svmRadial model, we used the sigest() function from the kernlab R package to calculate the range of the sigma hyperparameter. The cost hyperparameter was set to the following values: 0.25, 0.50, 2, 4, 8, 16, 32, 64, 128, 256, 512, and 1024. In all cases, the data were centered and scaled.

To interpret our models, we calculated the importance of each feature by utilizing the varImp() function from the caret R package. We then performed enrichment analysis for GO biological processes, KEGG and Reactome pathways, and disease ontology semantics using the clusterProfiler R package [34]. Last, we filtered the results with the following terms related to multiple myeloma: MAPK, RAS, RAF, MEK, ERK, ERK1, ERK2, PI3K, AKT, NF-KB, Jak-STAT, Wnt, Hedgehog, TNFa, mTOR, multiple myeloma, myeloid, leukemia, myeloma, Plasmacytoma, Amyloidosis, Chronic Lymphocytic Leukemia, Heavy Chain Disease, and Lymphoma [35,36].

## 3. Results

### 3.1. Task 1—Predicting the Stage of Multiple Myeloma

#### 3.1.1. Model Development for Disease Staging

We trained our models using the GSE6477 microarray dataset [37]. This dataset comprises 162 samples representing various stages of myeloma. Specifically, it includes 15 samples classified as Normal, 21 as MGUS (monoclonal gammopathy of undetermined significance), 23 as SMM (smoldering multiple myeloma), 75 as MM (newly diagnosed myeloma), and 28 as RMM (relapsed myeloma samples). We focused on 110 samples after excluding the SMM and RMM categories. We trained our models to separate the three classes: Normal, MGUS, and MM. The training process involved preprocessing the dataset, followed by splitting the data into training and validation sets. We used the training set to train the models and the validation set for hyperparameter tuning employing a tenfold cross-validation protocol repeated ten times. We used all other datasets (see Table 1) only for testing.

#### 3.1.2. Evaluation of Model Performance

**Figure 2:**
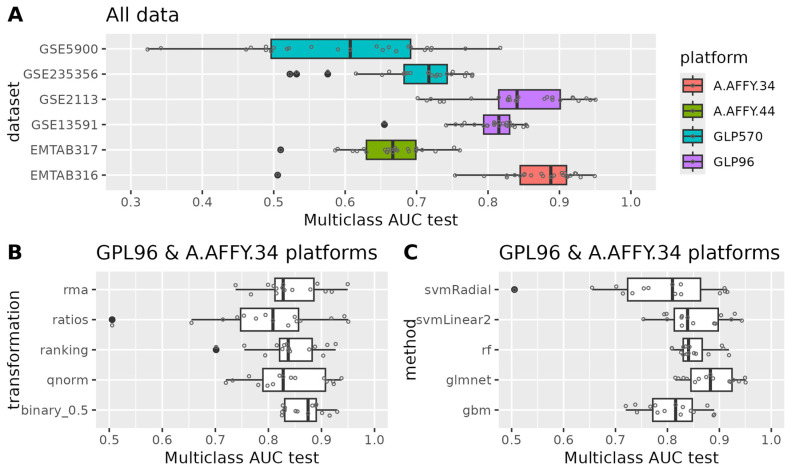
Models’ multiclass auc in the external validation sets. (**A**) The performance of the external dataset used across all data transformations and machine learning algorithms. (**B**) The relation of performance to the data transformations across datasets generated in GLP96 or A.AFFY.34 platforms and all machine learning algorithms. (**C**) The relation of performance to the machine learning algorithms across datasets generated in GLP96 or A.AFFY.34 platforms and all data transformations.

During training, all models consistently achieved a multiclass_auc with a cross-validation median ranging from 0.9 to 1 across various data transformations and machine learning methods (refer to Supplementary Figure S1). For the binary_0 transformation, where all expressions except from the lowest were set to 1, the median training performance of all models was around 0.5, which corresponds to a random classifier, as expected. Subsequently, we evaluated the multiclass_auc for all test datasets using all models and data transformations (see Figure 2 and Supplementary Figure S2).

Focusing on the platform of origin for the data, we noted that datasets (GSE13591, GSE2113) originating from the same platform (GPL96) as the training set exhibited similar multiclass_auc scores as seen during training (see Figure 2A). We observed a slight decline in performance, approximately 0.1 (see Supplementary Figure S3). EMTAB316 originated from A-AFFY-34, which is very close to GPL96, and showed similar multiclass_auc scores with training. In contrast, for datasets generated using different platforms (EMTAB317 from A-AFFY-44 and GSE5900, GSE235356 from GPL570), our models experienced a more significant decrease in performance. This suggests that performance variability across datasets may be attributed to differences in the platforms used for data generation. Regarding GSE235356, it is important to note that this dataset includes only MGUS and progressing MGUS samples, which could contribute to the observed decline in performance.

For the subsequent phases of our analysis, we focused on datasets generated from the GPL96 and A-AFFY-34 platforms. Concerning data transformations, we found that binary_0.5 yielded the highest multiclass_auc in the test datasets and exhibited less performance degradation compared to training results across all machine learning algorithms (refer to Figure 2B). Following binary_0.5, ranking, qnorm, rma, and ratio transformations were observed. In terms of machine learning algorithms, we observed that glmnet demonstrated the highest multiclass_auc in the test datasets across all data transformations and showed less performance degradation relative to the training phase (see Figure 2C). Succeeding glmnet, rf, svmlinear2, gbm, and svmRadial were observed.

#### 3.1.3. Model Interpretation

**Figure 3:**
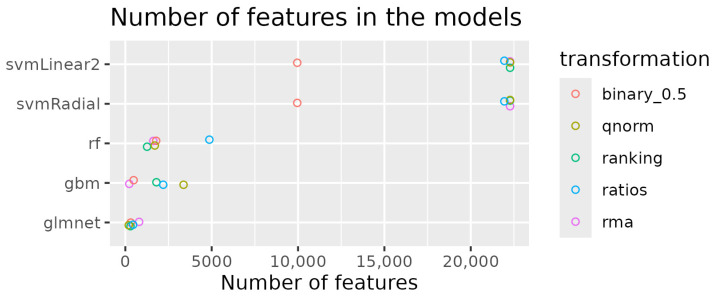
The number of features utilized by each model across different data transformations. The plot shows the variation in feature selection for each model, highlighting the range of features used in the analysis.

We calculated the importance of each feature for each model and data transformation combination. The svmLinear2 and svmRadial models utilized all available features (22,277). RandomForest models used between 1260 and 4866 features, while gbm models employed fewer features, ranging from 228 to 3371. The glmnet models used the least features, with counts between 197 and 798 (see Figure 3).

We observed that most selected features were specific to the model and transformation used. For the rma transformation, 72 probes were selected across glmnet, gbm, and rf models. Similarly, 62 probes, 58 probes, 101 probes, and 37 ratios were selected for the qnorm, ranking, binary_0.5, and ratio transformations, respectively (see Supplementary Figure S3A). Within the same model type (glmnet, gbm, rf), there was minimal overlap of probes across different normalizations. No probes overlapped across all five normalizations. The number of common probes for 4 out of 5 transformations was 8 for gbms, 30 for glmnet, and 31 for rf (see Supplementary Figure S3B).

**Figure 4:**
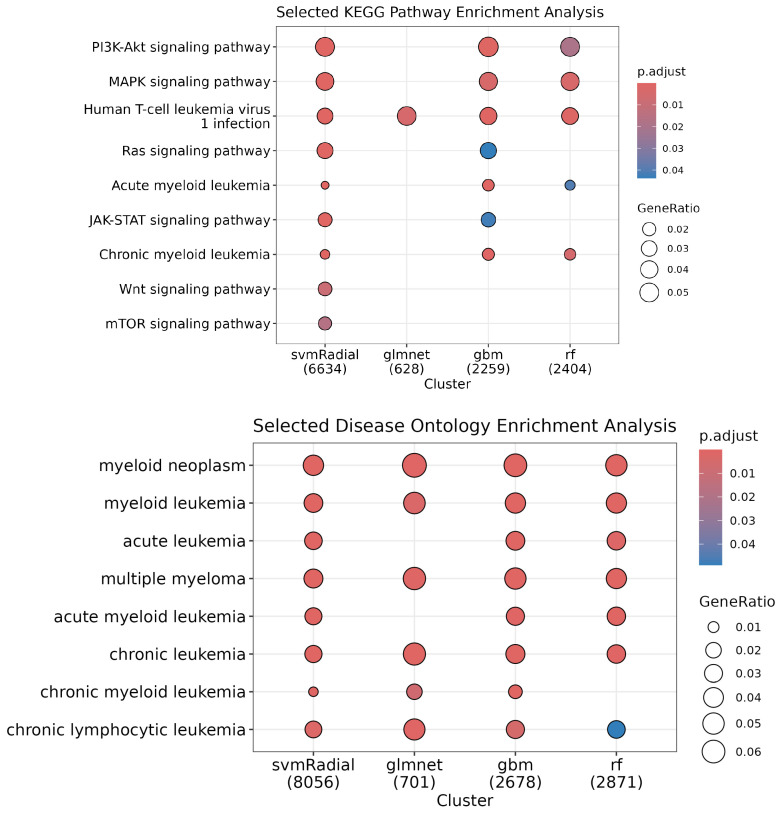
Enrichment analysis for the selected probes. (**Top**)**:** KEGG pathways associated with identified genes. This figure illustrates the KEGG pathways enriched for the genes identified by the machine learning models across different data transformations and training datasets. The pathways displayed are significantly associated with the probes selected by at least one model. Key pathways related to multiple myeloma, such as PI3K-Akt, MAPK, and Wnt signaling, are highlighted. (**Bottom**)**:** Disease-related terms associated with identified genes. The figure illustrates the distribution of disease-related terms associated with the genes identified by the models. The chart highlights how different methods and data transformations reveal connections to various cancers, including multiple myeloma. Each term represents a disease category. In both figures, the size and color indicate the strength of the association and statistical significance.

Next, using the probes selected by at least one data transformation for each method, we performed enrichment analysis of biological processes via Gene Ontology (GO) terms (see Supplementary Figure S4), Reactome pathways (see Supplementary Figure S5), KEGG pathways (see Figure 4), and disease ontology semantics (see Figure 4). Our models identified probes whose respective genes are involved in pathways highly related to multiple myeloma, such as the PI3K-Akt, MAPK, JAK-STAT, and Wnt signaling pathways, the RAF/MAP kinase cascade, NF-kB-related pathways, and signaling by RAS and BRAF mutants. The disease ontology semantics highlighted several blood cancers, including multiple myeloma, across all methods.

### 3.2. Task 2—Predicting Progression from MGUS to MM

#### 3.2.1. Model Development for Disease Progression Prediction

We trained our models employing individual datasets—GSE235356, GSE6477, and EMTAB317—and combining datasets generated from the GLP96 and A-AFFY-33 platforms in one dataset, specifically GSE6477, GSE2113, EMTAB316, and GSE13591. For the GSE235356 dataset [27], we focused on training models to distinguish between *MGUS and progressing MGUS*, where the latter refers to MGUS cases that progressed to MM. We used 10-fold cross-validation to optimize hyperparameters and employed a 10-fold nested cross-validation protocol to evaluate model performance. Due to the computational expense of nested cross-validation, we optimized hyperparameters using standard 10-fold cross-validation instead of performing 10 repeated iterations. We chose nested cross-validation for performance evaluation because we did not have an external dataset containing both MGUS and progressing MGUS samples. In all other scenarios, we trained the models to differentiate between *MGUS and MM*, optimizing hyperparameters with 10-fold cross-validation repeated 10 times, as in Task 1. For consistency, we applied the same machine learning models and data transformations as in Task 1 (see Figure 1).

The scope of these two training approaches (*MGUS* vs. *progressing MGUS* and *MGUS* vs. *MM*) was twofold. First, we aimed to assess whether models trained to differentiate MGUS from MM could effectively distinguish MGUS from progressing MGUS, using the GSE235356 dataset for testing. Second, we sought to compare the performance of these models with those specifically trained to separate MGUS from progressing MGUS patients. This comparison would provide insights into whether models generalized well across related conditions or if specialized training was required for optimal performance in predicting MGUS progression.

#### 3.2.2. Evaluation of Model Performance

**Figure 5:**
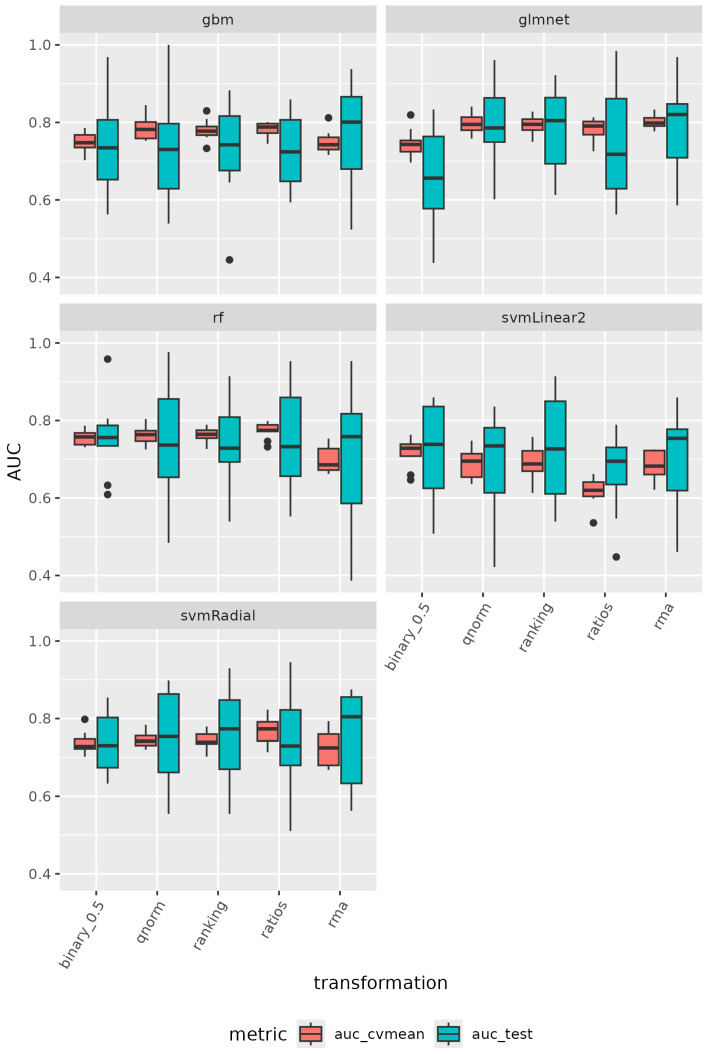
Performance of machine learning algorithms on the GSE235356 dataset. The figure displays the distribution of the mean cross-validation AUC (auc_cvmean, shown in red) and the distribution of the AUC from the outer hold of the nested cross-validation (auc_test, shown in cyan) for each algorithm when the GSE235356 dataset was used for training and testing. The auc_cvmean represents the performance across the cross-validation folds, while the auc_test indicates the model’s generalizability on unseen data. The comparison of these distributions highlights the algorithm’s generalization and stability.

**Figure 6:**
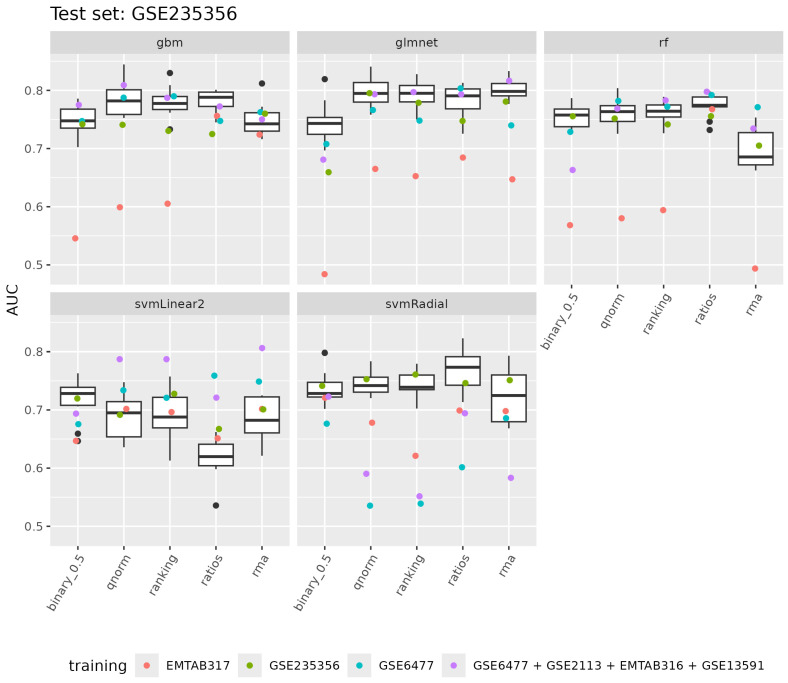
Model performance in differentiating MGUS from progressing MGUS across different datasets. The boxplots show the distribution of the mean cross-validation AUC for models trained to differentiate MGUS from progressing MGUS using the GSE235356 dataset. The colored points represent the performance of each algorithm–data transformation combination across various training datasets: models trained with the EMTAB317 dataset are shown in red; those trained with the GSE235356 dataset are in green; models trained with the GSE6477 dataset are shown in cyan; and those trained with the combined GSE6477 + GSE2113 + EMTAB316 + GSE13591 datasets are depicted in purple. Notably, in all cases except for the second (GSE235356), the models were specifically trained to distinguish MGUS from MM.

Using the GSE235356 dataset for training, we calculated the cross-validation area under the ROC curve (AUC) during model optimization (auc_cv), the mean cross-validation AUC (auc_cvmean), and the outer cross-validation AUC from the nested cross-validation protocol (auc_test). Among the models, glmnet achieved the best performance, followed by gbm, rf, svmRadial, and svmLinear2 (refer to Figure 5). Specifically, glmnet with rma, qnorm, or ranking transformations showed the highest performance, with both auc_cvmean and auc_test around 0.8 (refer to Figure 5). All algorithms and data transformations also demonstrated good generalization performance in the outer cross-validation fold. The mean auc_test across all outer cross-validation folds fell within the AUC distribution achieved during training cross-validation (refer to Figure 6).

We trained our models to distinguish *MGUS from MM* using the GSE6477 dataset. These models achieved a training cross-validation AUC median ranging from 0.93 to 1 across all data transformations and machine learning methods (refer to Supplementary Figure S6). Most models demonstrated good generalization performance when applied to other datasets (EMTAB316, EMTAB317, GSE13591, GSE2113) for identifying *MGUS from MM* (refer to Supplementary Figure S7). For EMTAB316 and GSE2113, the test AUC median was 0.9 and 0.86 across all data transformations and machine learning methods. For GSE13591, the test AUC median was 0.8 across all methods and transformations. The models achieved the lowest test AUC for the EMTAB317 dataset, with an AUC median of 0.7. This result is consistent with our findings in task 1 and likely due to the different microarray platforms used to generate the data.

When we applied our models to separate *MGUS from progressing MGUS*, the models differentiated the two classes. Specifically, the test AUC achieved by gbm, glmnet, rf, and svmLinear2 ranged from 0.7 to 0.8, falling within the AUC distribution achieved with cross-validation during training with the GSE235356 dataset (refer to Figure 6) and within the outer cross-validation auc_test distribution of the GSE235356 dataset (refer to Supplementary Figure S11). In the case of svmRadial, the auc_test ranged from 0.54 to 0.69; in all cases except rma normalization, it was below the training AUC cross-validation distribution but inside the outer cross-validation auc_test distribution of the GSE235356 dataset.

Next, we trained our models to distinguish *MGUS from MM*, employing the EMTAB317 dataset. These models achieved a training cross-validation AUC median ranging from 0.79 to 0.94 across all data transformations and machine learning methods (refer to Supplementary Figure S8). Most models demonstrated good generalization performance when applied to other datasets (EMTAB316, GSE13591, GSE2113, GSE6477) for identifying *MGUS from MM* (refer to Supplementary Figure S9). For EMTAB316 and GSE6477, the median test AUC was close to 0.75 and 0.82 across all data transformations and machine learning methods. For GSE13591 and GSE2113, the median test AUC was close to 0.86 and 0.91 across all methods and transformations.

When we applied our models to separate *MGUS from progressing MGUS*, the models showed a test AUC performance ranging from 0.5 to 0.76, with a median of 0.65. The test AUC achieved by gbm, glmnet, rf, and svmRadial fell below the cross-validation AUC distribution achieved during training with the GSE235356 dataset (refer to Figure 6) but within the outer cross-validation auc_test distribution of the GSE235356 dataset (refer to Supplementary Figure S11). Interestingly, for svmLinear2, the test AUC fell within the AUC cross-validation distribution during training with the GSE235356 dataset for all data transformations.

Last, we trained our models to separate *MGUS from MM* using all datasets generated from the GLP96 or A-AFFY-33 platforms (*GSE6477 + GSE2113 + EMTAB316 + GSE13591*). Our models achieved a training cross-validation AUC median ranging from 0.94 to 0.97 across all data transformations and machine learning methods (refer to Supplementary Figure S10). Similarly, we applied our models to separate *MGUS from progressing MGUS*. The models’ test AUC performance ranged from 0.55 for svmRadial using ranking to 0.82 for glmnet employing rma, with a median performance across all models and data transformations of 0.77. Importantly, the test AUC achieved by gbm, glmnet, and rf fell within the cross-validation AUC distribution achieved during training with the GSE235356 dataset (refer to Figure 6) and within the outer cross-validation auc_test distribution of the GSE235356 dataset (refer to Supplementary Figure S11). For svmRadial, the test AUCs fell below the cross-validation AUC distribution for all data transformations except binary_0.5, and at the lower end of the outer cross-validation auc_test distribution. Interestingly, for svmLinear2, the test AUC fell above the cross-validation AUC distribution for all data transformations except binary_0.5, and on the upper end of the outer cross-validation auc_test distribution. Additionally, with the inclusion of the GSE2113, EMTAB316, and GSE13591 datasets, glmnet and svmLinear2 showed a 0.05 increase in median test AUC across all data transformations compared to when only the GSE6477 was used for training; however, these differences were not statistically significant.

Next, we conducted a permutation test to assess the statistical significance of the observed model performances in the test dataset in comparison to a random classification. In this analysis, we permuted the class labels (*MGUS, progressing MGUS*) and recalculated the auc_test for each model. Models with auc_test values close to 0.5, which correspond to a random classifier, did not demonstrate a statistically significant different performance from random, as expected. Conversely, models with auc_test values exceeding 0.7 showed highly significant results, clearly falling outside the permutation distribution (refer to Supplementary Figure S12).

#### 3.2.3. Model Interpretation

We focused on interpreting the models trained using either the GSE235356 dataset or all GPL96 datasets combined. The svmLinear2 and svmRadial models utilized all available features. When all GPL96 datasets were used for training, the rf models employed between 2169 and 10,269 features, glmnet selected between 236 and 859 probes, and gbm chose between 214 and 685 probes. In contrast, when the GSE235356 dataset was used for training, the rf models utilized between 3090 and 11,650 features, glmnet selected between 10 and 792 probes, and gbm chose between 47 and 2084 probes (see Supplementary Figure S13). We also assessed the overlap of probes selected across the two training datasets. For gbm and glmnet, only a small number of probes (ranging from 1 to 99) were selected in both cases. In contrast, the rf models showed a higher degree of overlap, with common probes ranging from 482 to 5459 (refer to Supplementary Figure S14).

**Figure 7:**
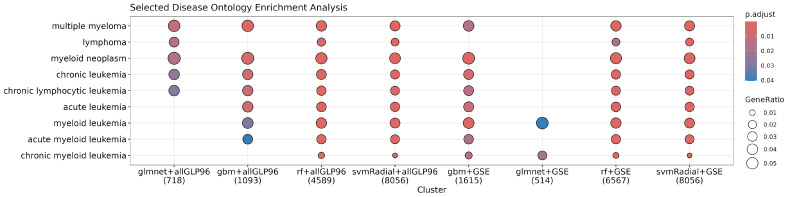
Disease-related terms associated with identified genes. The figure illustrates the distribution of disease-related terms associated with the genes identified by the models. The chart highlights how different methods across all data transformations and the different training datasets reveal connections to various cancers, including multiple myeloma. Each term represents a disease category. The size and color indicate the strength of the association and statistical significance. “all GLP96” refers to the combined dataset of GSE6477 + GSE2113 + EMTAB316 + GSE13591, and “GSE” to the GSE235356 dataset.

Using the probes selected by at least one data transformation for each method and training dataset, we conducted enrichment analyses on Gene Ontology (GO) biological processes, KEGG pathways, Reactome pathways, and disease ontology semantics. The analysis revealed that our models identified probes associated with genes involved in pathways closely related to multiple myeloma, such as PI3K-Akt (see Supplementary Figure S17), MAPK (see Supplementary Figures S15–S17), Wnt signaling (see Supplementary Figure S16), BRAF and RAF1 fusion signaling (see Supplementary Figure S17), and mTOR pathways (see Supplementary Figure S16). The disease ontology analysis also underscored the relevance of several blood cancers, including multiple myeloma, across most methods and training datasets (see Figure 7).

## 4. Discussion

In this study, we utilized machine learning (ML) techniques to tackle two critical challenges in multiple myeloma (MM): *predicting the disease stage* and *predicting disease progression* from monoclonal gammopathy of undetermined significance (MGUS) to MM. Through comprehensive data preprocessing, model training, and evaluation across multiple datasets, we aimed to enhance diagnostic precision and offer valuable prognostic insights for hematologic malignancies.

The first focus of our study was on predicting the stage of MM. Accurate staging is crucial for determining the appropriate treatment strategy and prognosis. We developed models using various ML algorithms, including ElasticNet, Random Forest, Boosting, and Support Vector Machines. These models were trained on a dataset comprising samples from different stages of MM and healthy samples, and their performance was evaluated on external validation datasets. The multiclass area under the curve values obtained during cross-validation and testing consistently demonstrated that the selected features and ML algorithms effectively capture the biological differences across disease stages. Specifically, our models identified genes involved in pathways that are well documented in the literature for their roles in MM pathogenesis (see below for details). Among the models evaluated, gbm achieved the highest performance in training, and glmnet showed minimal degradation across different data transformations and datasets, indicating its robustness and generalizability. Our findings align with the growing body of literature that supports the use of ML in oncology, particularly in hematologic malignancies. Previous studies have shown the effectiveness of ML algorithms in improving diagnostic accuracy and risk stratification in MM [26,38]. The variability in model performance across different platforms, observed in datasets from GPL96, A-AFFY-34, GPL570, and A-AFFY-44, underscores the challenges of integrating data from diverse sources. This issue has been documented in the literature, where differences in data generation methods significantly affect model performance [39,40].

Predicting the progression of monoclonal gammopathy of undetermined significance (MGUS) to multiple myeloma (MM) remains one of the most pressing challenges in managing plasma cell disorders. The early identification of high-risk MGUS patients could significantly enhance clinical outcomes by enabling timely interventions that might delay or even prevent the onset of MM. A significant obstacle in this effort is the limited availability of datasets that include progressing MGUS patients, as these cases are inherently rare and difficult to procure. To address this challenge, we employed a two-pronged approach. First, we developed machine learning models using a dataset specifically containing *MGUS and progressing MGUS* patients, achieving a maximum AUC of 0.8 with the glmnet model combined with quantile normalization. Other models and data transformations demonstrated good generalization performance, with AUC values around 0.75. This result highlights the potential of machine learning in identifying high-risk MGUS patients even with limited data availability. Second, to evade the scarcity of progressing MGUS samples, we trained our models using multiple datasets containing both *MGUS and MM* patients. These models were then evaluated for their ability to distinguish *MGUS from progressing MGUS* cases. Our findings indicate that machine learning models, including ElasticNet, Boosting, SVM with linear kernel, and Random Forest, achieved AUC values close to 0.8, suggesting a strong potential for these models in risk stratification. Although some models, such as SVM with radial kernel, demonstrated lower performance, the overall results underscore the utility of incorporating both MGUS and MM data in predictive modeling.

To our knowledge, this study is the first to develop comprehensive machine learning models specifically designed to predict the progression of MGUS to MM by leveraging datasets from both MGUS and MM cases. Our innovative approach of integrating MM data to train models that predict MGUS progression offers a novel and potentially more accurate method for risk assessment. This methodology could have significant clinical implications, particularly in distinguishing MGUS patients who require closer monitoring from those who may not. The novelty and potential impact of our approach are further emphasized by recent reviews in the field, such as the one by Awada et al. [41], which highlight the need for more sophisticated predictive models that integrate data across disease stages to enhance prognostication and treatment planning.

The feature selection and enrichment analyses conducted in this study provided significant insights into the molecular pathways and biological processes involved in the progression of multiple myeloma. Our models consistently identified genes involved in critical signaling pathways, such as PI3K-Akt, MAPK, Wnt, and mTOR. These pathways are well known for their roles in cell growth, survival, and proliferation, and their involvement in MM pathogenesis is well documented [42]. For instance, the PI3K-Akt pathway has been widely recognized as a key player in MM, influencing proliferation, migration, apoptosis, and autophagy [43]. Similarly, the MAPK pathway is involved in the regulation of cell proliferation, survival, and differentiation, and its dysregulation has been implicated in various cancers, including MM [44,45]. The Wnt pathway, which is crucial for cell differentiation and proliferation, has also been associated with MM progression, particularly in the context of bone disease [46]. The consistency of our results with established biological knowledge validates our models and suggests potential therapeutic targets that could be explored in future research.

While the results of this study are promising, several limitations should be considered when interpreting our findings. One significant challenge is the variability in model performance across different microarray platforms. This variability suggests a need for more comprehensive cross-platform validation to ensure the robustness of our models when applied to data generated from various microarray platforms, which may have different processing methods and platform-specific characteristics. Ensuring model performance across these platforms is crucial for the generalizability of our models in clinical settings. Moreover, the relatively small number of datasets used in this study and the focus on a limited set of machine learning algorithms may have constrained our ability to explore other potentially valuable approaches. Future research should aim to include a more extensive variety of datasets, especially those generated from different omics technologies (e.g., proteomics, genomics, and transcriptomics), to enhance the generalizability and robustness of the models. This approach would help address the limitations of relying solely on microarray data and provide a more comprehensive understanding of disease mechanisms. Additionally, integrating clinical data, such as patient demographics and treatment history, could provide a more comprehensive understanding of disease progression and improve the clinical applicability of the models. These challenges are well recognized in the literature [47,48,49]. All studies emphasize the need for cross-platform validation and standardization in ML models, particularly in the context of precision medicine, where the ability to generalize across different datasets is crucial for clinical implementation.

## 5. Conclusions

This study demonstrated the utility of machine learning models in addressing two critical challenges in multiple myeloma (MM): accurate disease staging and predicting the progression of monoclonal gammopathy of undetermined significance (MGUS) to MM. By leveraging diverse datasets and ML algorithms, we achieved robust performance, with ElasticNet and Boosting models consistently yielding high AUC values for both tasks. Importantly, feature selection identified key signaling pathways central to MM pathogenesis, aligning with established biological knowledge and suggesting potential therapeutic targets. While promising, our findings highlight the need for broader dataset inclusion, cross-platform validation, and the integration of clinical data to further enhance the models’ generalizability and clinical applicability. These advances could pave the way for more precise prognostic tools and targeted interventions in hematologic malignancies. Also, to improve model robustness, an ensemble classification approach could be explored. By combining multiple machine learning algorithms, ensemble methods can reduce performance variability across platforms and enhance prediction accuracy, offering more reliable generalizability for clinical use.

## Figures and Tables

**Table 1:** The number of samples per dataset and disease stage. The table is sorted by the total number of samples. The empty cells correspond to a zero number of samples.

Platform	Dataset	Normal	MGUS	Progressing MGUS	MM	Total Number of Samples
GLP96	GSE2113		7		39	46
A-AFFY-33 A-AFFY-34	EMTAB316		7		65	72
GLP570	GSE5900	22	44			66
GLP96	GSE6477	15	22		73	110
GLP96	GSE13591	5	11		133	149
A-AFFY-44	EMTAB317		23		226	249
GLP570	GSE235356		319	39		358
**Total**	7	42	433	39	536	1050

## Data Availability

The datasets analyzed in this study are available in GEO and ArrayExpress repositories under the following accession numbers: (a) GEO: GSE2113, GSE5900, GSE6477, GSE13591, GSE235356, (b) ArrayExpress: EMTAB316, EMTAB317.

## Supplementary Materials

The following supporting information can be downloaded at https://www.mdpi.com/article/10.3390/cancers17020332/s1. Figure S1: Distribution of Multiclass AUC from Ten-Fold Repeated Cross-Validation; Figure S2: Performance of Machine Learning Model-Data Transformation Combinations on External Datasets; Figure S3: Number of Common Probes Selected; Figure S4: GO Enrichment Analysis of Identified Genes; Figure S5: Reactome Pathways Enrichment Analysis of Identified Genes; Figure S6: Distribution of AUC from Ten-Fold Repeated Cross-Validation; Figure S7: Performance of Machine Learning Model-Data Transformation Combinations on External Datasets; Figure S8: Distribution of AUC from Ten-Fold Repeated Cross-Validation; Figure S9: Performance of Machine Learning Model-Data Transformation Combinations on External Datasets; Figure S10: Distribution of AUC from Ten-Fold Repeated Cross-Validation; Figure S11: Model performance in differentiating MGUS from progressing MGUS across different datasets; Figure S12: Permutation Testing; Figure S13: Feature Utilization Across Models and Data Transformations; Figure S14: Number of Common Probes Selected Across Training Datasets; Figure S15: Enrichment Analysis of Selected Gene Ontology (GO) Biological Processes; Figure S16: KEGG Pathways Associated with Identified Genes; Figure S17: Reactome Pathways Enrichment Analysis of Identified Genes.
